# Supplementary material for: BiG-SLiCE: A highly scalable tool maps the diversity of 1.2 million biosynthetic gene clusters
Source: Gigascience. 2021 Jan 13;10(1):giaa154. doi: 10.1093/gigascience/giaa154 (PMC7804863; doi:10.1093/gigascience/giaa154)
Supplement: giaa154_Response_to_Reviewer_Comments_Original_Submission [file giaa154_response_to_reviewer_comments_original_submission.pdf]

Author's Response To Reviewer Comments

Close

General remarks
We thank the reviewers for providing many insightful comments and useful feedback on our manuscript and software (including its documentation), and for expressing their encouragingly positive remarks on our work. We wrote a point-by-point answer to each of the reviewer's comments and made some modifications to the manuscript and figures accordingly. To summarize:
1. We fixed all broken links in our GitHub documentation and added an explicit description of how to use the provided input folder template for doing a "minimal test run" of BiG-SLiCE. We also added some extra instruction on programmatic access to BiG-SLiCE output and on how to generate a custom but valid antiSMASH5-formatted GenBank file to be used as an input in BiG-SLiCE.
2. We improved the clarity of the manuscript by rephrasing some sentences related to the points in the comments.
3. We added a previously overlooked discussion on earlier-published BGC vectorization methods. We also now mention a number of well-known alternative BGC prediction algorithms besides antiSMASH.
4. We extended the future perspectives discussion to express our interest in taking BiG-SLiCE beyond its current antiSMASH/MIBiG-bound coverage. We also took the chance to add a paragraph describing the potential of the sub-Pfam approach outside the scope of GCF analysis.
Reviewer #1 points
(1) One of the key points in the work is the approach to transform BGCs into vectors, a process that is detailed with precision and makes good sense. But the authors seem to overlook other (perhaps more straight forward) vectorisation methods such as BioVec (10.1371/journal.pone.0141287) or Pfam2vec (10.1093/nar/gkz654). The use of such approaches could reduce the dimensions of the vectors and potentially speed up the actual clustering step. Furthermore, Pfam2vec authors have shown that averaging the vectors of proteins in BGCs provides useful representations for clustering.
Response:
Actually, we saw our method as a simplistic, more straightforward approach compared to the aforementioned methods. BioVec can be seen as an alternative approach to pHMMs in the context of modeling protein sequence identity. Although we can similarly extend BioVec's protein features (termed ProtVec) to multi-protein sequences (i.e., BGCs) to directly get a vector representation of our data, we have not yet seen the method's applicability (or practicality) compared to pHMM-based Pfam assignment, which has been the workhorse of BGC detection algorithms like antiSMASH, PRISM (10.1093/nar/gkx320) and the likes. In BiG-SLiCE, we semi-manually curate a more sensitive set of pHMM models (the sub-Pfams), and then use their ranked bitscores as a proxy for sequence-level identity.
With Pfam2Vec, context (i.e., biological meaning) of each protein family in the (full) Pfam database is computationally inferred via Recurrent Neural Network-based "words" embedding, with a training corpus typically coming from selected thousands of genomes. As mentioned in a related paper (10.1101/524280), the construction of this Word2Vec-derived model involves extensive hyperparameter tuning. In BiG-SLiCE, we took a simpler "semi-supervised" route by manually filtering and generating the Pfam models relevant for secondary metabolite biosynthesis, practically assigning a human-curated context to each of the selected models. This will avoid unintended bias caused by false correlation, such as associating ABC transporters with RiPP clusters, a limitation mentioned in the original DeepBGC article.
Indeed, normally, the high dimensional (~6,000) feature matrix of BiG-SLiCE might present a challenge for clustering algorithms such as K-means(++); a well-known problem known as the "curse of dimensionality" (10.1126/science.153.3731.34). However, in our case, the sparse but context-specific nature of the extracted BGC vectors allows a linear, greedy algorithm like BIRCH to cluster the BGCs in an efficient manner (clustering time average of four hours for 800,000 BGCs; compared to the ~100 hours of the pHMM scanning step).
Last but not least, the current implementation gives a clear and explainable representation of BGC and GCF models, i.e., we can almost trace back the exact (sub-Pfam) domain composition of BGCs and GCFs via their feature values. This is contrary to the embedded vectors of both ProtVec and Pfam2Vec, which work well when combined with, e.g., deep learning algorithms, but can be hard to interpret for exploratory purposes.

We have been aware of these approaches during the design phase of BiG-SLiCE, but indeed overlooked them in our writing. We have now added a paragraph discussing ProtVec and Pfam2Vec approaches in our “BGC feature extraction” subsection and an additional forward-looking perspective in our “Conclusions and Future Perspectives” subsection.
(2) Input folder: Even though the wiki page about the input folder is very useful, a downloadable example of an input folder will make it very easy to test and users can familiarize with the output of the tool.
Response:
We already had a downloadable input folder example in our GitHub repository (https://github.com/medema-group/bigslice/tree/master/misc/input_folder_template). We nowspecifically mention this folder for a “minimal test run” on our README page.
(3) README file (Quick start / 3. Fetch the latest HMM models): The name of the script in the instruction is "download_bigslice_hmmdb.py" while the installed script does not have the ".py" extension.
Response:
We have fixed the specified typo on our README page.
(4) The minimum information that a cluster's genbank file must contain to be processed must be provided. Meaning, which features/annotations should the file have?. This will be helpful when an alternative tool to antiSMASH is used to detect BGCs, or in cases where the cluster borders are manually refined.
Response:
At this moment, we rely on curation-based tools and databases such as antiSMASH and MIBiG in order to tune our feature extraction and algorithm on well-studied BGC classes. Technically speaking, we rely on the output format from antiSMASH and MIBiG to assign BGC (sub-)classes and to separate (putatively) complete and fragmented BGCs. In the future, we plan to accept file formats produced by other, more “exploratory” tools and databases. We have added some extra sentences in our “Conclusions and Future Perspectives” to communicate this to readers. AntiSMASH cluster GenBank files with manually-refined cluster borders will be accepted by BiG-SLiCE as inputs without any extra requirements. Furthermore, to support people who want to use their own custom GenBank file inputs, we have now added an adaptor script (https://github.com/medema-group/bigslice/tree/master/misc/generate_antismash_gbk) along with a short instruction in our README page (section: “Custom GenBank input”) on how to transform a regular GenBank file into a valid antiSMASH5-like cluster GenBank file suitable for use in BiG-SLiCE.

Reviewer #2 points
(1) BiG-SLICE is quite rigidly designed around input data coming (exclusively) from AntiSMASH (only seems to accept antismash-modified GenBank files); we could not get it to accept more standard GenBank files or ones generated by other tools such as DeepBGC.
Response:
Indeed, for its initial release, we limited BiG-SLiCE usage around curation-based tools and databases like antiSMASH and MIBiG, as we wanted to be able to carefully optimize and benchmark its feature extraction strategy (i.e., the biosynthetic- & sub-Pfams) and clustering algorithm (i.e., using antiSMASH’s & MIBiG’s metadata to separate complete and fragmented BGCs) for well-studied, experimentally-supported BGC classes. Looking into the future, it is perfectly viable (and under our consideration) to expand its coverage for other, more “exploratory” tools and databases like DeepBGC. We have added some extra sentences in our “Conclusions and Future Perspectives” to communicate this to readers. To support for people who want to use their own custom GenBank files, we have now added an adaptor script (https://github.com/medema-group/bigslice/tree/master/misc/generate_antismash_gbk) along with a short instruction in our README page (section: “Custom GenBank input”) on how to transform a regular GenBank file into a valid antiSMASH5-like cluster GenBank file suitable for use in BiG-SLiCE.
(2) BiG-SLICE results are all stored in the SQL database and export is not straightforward. Programmatic access to the database is not (well) documented either. A more flexible design for BiG-SLICE, in particular export of results as flat-text files for easy import to enable quick follow-up analysis would be highly desirable. I feel that while GUI access may be most important for tools such as AntiSMASH, most users interested in really large-scale BGC analyses will (have to) possess more advanced computational skills and would therefore likely value programmatic access over graphical outputs. As a side note, I'd encourage the authors to consider more light-weight designs for future versions of BiG-SLICE as these may also scale better. Fig 5A to me suggests that further gains in efficiency (necessary for ever larger analyses of MAGs etc) cannot be expected from algorithmic improvements (clustering currently only takes ~10% of the whole runtime), but from improved software engineering (e.g. for leaner IO).
Response:
One key reason to center our design around SQL-based data storage is so that BiG-SLiCE can target

both advanced and basic users at the same time.
We envision that BiG-SLiCE will be used to process at least double the amount of ~1.2M BGCs in the manuscript, especially if we want to consider taking inputs from more ‘exploratory’ BGC detection tools like DeepBGC that predict larger numbers of clusters. For that, SQL(ite)-based data storage provides a simple yet powerful way to process that data for downstream analyses (including generating any custom tabular and text-based files on demand) by advanced users. In the paper’s supplementary data, we give some example SQL queries and python scripts that can be reused to familiarize users with programmatic access to BiG-SLiCE data. We have now made the instruction clearer in our README page (section: “Programmatic Access and Postprocessing”). In the future, we plan to make a more comprehensive wiki and guideline on how to perform such operations, as we are aware that although starting to gain increased adoption in the field, SQL is not something everybody is already familiar with from the start. Furthermore, the same SQL database will support flexible development of even more extensive and/or customized features on top of its Flask-based user interface engine. Although the one-time clustering process can indeed be performed by (slightly) more advanced users, its resulting output can be hosted locally (i.e., on an intranet), so that regular users can access it via their web browsers. We find that this automated visualization strategy is important to enable a detailed exploration / expert-based interpretation of the large-scale clustering results and would be appreciated by both target audiences. We give an example of this “scenario” in our newly online BiG-FAM database (https://bigfam.bioinformatics.nl/, 10.1093/nar/gkaa812), which was built upon BiG-SLiCE’s SQLite and flask-based output.
As for improving BiG-SLiCE’s runtime efficiency, although there is a lot to gain over its current SQL-based implementation (i.e., at this moment, all SQL operations are naively done in series, which can be significantly improved upon taking a more sophisticated parallelization strategy), we do not see throwing the architecture away in favor of, e.g., tabular-based file approach as a good trade-off due to reasons described above.
(3) https://github.com/medema-group/bigslice: hyperlink to pre-processed result on ~1.2M microbial BGCs from the NCBI database produces 404
Response:
We have fixed the broken link on our README page.
(4) https://github.com/medema-group/bigslice/wiki/Input-folder: GTDB-toolkit and NCBI taxonomy script hyperlinks produce 404
Response:
We have fixed the broken link on our README page.
(5) https://github.com/medema-group/bigslice/wiki/Program-parameters: program parameters are not documented
Response:
We have now put up the explanation of each program parameters on our wiki page.
(6) The `bigslice --version` command does not print the version.
Response:
Due to the tricky behavior of python argparse, the previous version of BiG-SLICE required an input folder to be specified (in this case “bigslice --version .”; mind the extra dot). We have now implemented a workaround to enable the mentioned command to correctly print the software version (will only be available through PyPI on the next stable release).
(7) Page 1. The sentence "Due to the sheer size of microbial and enzymological biodiversity, there exists a vast repertoire of potentially useful compounds remains to be unearthed" needs rewording.
Response:
We understand that this sentence might be too wordy and hard to follow. We have rephrased it into a simpler and clearer one.
(8) Page 5. Fig 2C: Separation between distributions could be quantified by receiver-operating-characteristic (ROC) curves (also applies to Fig 3). Using a varying distance cutoff, pairs from the same class above the cutoff are counted as true-positives, below the cutoff as false negatives; pairs from different classes above the cutoff as false positives and as true negatives below the cutoff.
Response:
We do not intend to make the underlying questions a classification problem. In Figure 2C, BGCs can have multiple classes, and even within the same antiSMASH class, the domain content (and thus the observed distance) can vary. In Figure 3, although we can say that the MIBiG groups can serve as a “ground truth”, the manually described families should only be taken as a reference (to objectively judge BiG-SLiCE results) rather than as a classifier benchmark.
(9) Fig 2D: consider changing scale to 0-100 for "percent" identity, as I am assuming this is referring to 0-100% and not 0-1.0%.
Response:
The left y-axis shows Pearson correlation values (R), as opposed to %-identity. We thought the title and

its coloring might mislead readers, thus we have now modified the text accordingly.
(10) Page 13. Why is the T = 1100, established to be optimal in Fig 3A, not included among the thresholds tested here?
Response:
Although we picked T=1,100 as an example for Figure 3 due to their similarity in the number of GCFs compared to manually curated data (while still maintaining a relatively high V-score), we do not want to suggest that this threshold is universally optimal and should be used for the full-scale dataset. When running the full analysis, we chose a series of arbitrary thresholds and showed how they can be used in tandem to explore and interpret the data (e.g., in Figure 8). We have reworded the corresponding main text and caption of Figure 3A to avoid this confusion.
(11) Page 14. Why was k = 500 chosen for k-means clustering?
Response:
This was picked (somewhat arbitrarily) to generate a phylogram that is rich in information, but small enough that it will still be clearly visible for the printed paper figure.
(12) Is the longish explanation for interpretation of non-deterministic k-means clustering needed? Why not stick to hierarchical clustering down to the 120K GCFs instead?
Response:
A pragmatic answer: runtime and memory cost. Furthermore, the long explanation can also help to further instill the correct mindset when looking at (BiG-SLiCE’s) clustering in general: that each result (from different thresholds) should be treated as a one-sided view of the complete object and that there is no single, universal “true” value - as with all clustering problem.
(13) Page 15. Fig 6: Why aren't fungal clades clustered together (despite mentioned distinctions from prokaryotic clades)? Dendrogram in the middle is a bit too small to be used for interpretation.
Response:
The phylogram depicts functional (and by proxy, chemical) rather than taxonomy diversity. In this case, although fungal PKS BGCs clade together, they are still functionally distant from the (non-hybrid) fungal NRPSes, as the set of domains they shared will differ significantly, as opposed to, e.g., bacterial PKSes.
(14) Page 18. Fig 7: Consider labeling the x-axis (not just in the caption)
Response:
We had placed a label for the x-axis “d (x100)” in the lower right corner of the plot. We have now adjusted the text in the figure to make it clearer for the readers.

Close
