# Supplementary material for: BiG-SLiCE: A highly scalable tool maps the diversity of 1.2 million biosynthetic gene clusters
Source: Gigascience. 2021 Jan 13;10(1):giaa154. doi: 10.1093/gigascience/giaa154 (PMC7804863; doi:10.1093/gigascience/giaa154)
Supplement: giaa154_Reviewer_1_Report_Original_Submission — Santiago Fragoso -- 10/1/2020 Reviewed [file giaa154_reviewer_1_report_original_submission.pdf]

Reviewer Report

Title: BiG-SLiCE: A Highly Scalable Tool Maps the Diversity of 1.2 Million Biosynthetic Gene Clusters

Version: Original Submission **Date:** 10/1/2020

Reviewer name: Santiago Fragoso

Reviewer Comments to Author:

The paper and software are very interesting. The work provides an alternative tool (fast and sound) to analyse the large diversity of biosynthetic gene clusters. The visualisation engine is simple and intuitive. Also, detailed information can be gathered from it. Throughout the paper, the limitations of the tool and approach are clearly stated.

The paper presents a tool for clustering biosynthetic gene clusters into families. The method archives fast results by transforming the set of proteins on a BGC into a vector, based on the absence/presence and bitscores of hits against a pHMM library. These BGC representations allow clustering using the BIRCH algorithm, which is fast. The authors build a use case of the tool using a very large dataset of publicly available BGCs to assess the global diversity of BGCs across taxonomy. They provide the output of their results, allowing future users to use this as a reference set of Gene Cluster Families.

The rationale, methodology, and results of the method and the use case are clearly presented throughout the paper. The implementation of the method is well documented for minimal use and the software seems to work well. The display of the information in the visualisation engine is quite good.

The main contribution of the paper is to provide a new and fast tool for large scale downstream analysis of BGCs, which is very welcome. Currently, discovery tools, such as antiSMASH and deepBGC, offer a limited set of categories in which the putative BGCs can be classified. This results in many of the publications that include BGC discovery in large scale datasets restricting their analysis to BGC-classes counts by clades/biomes and presenting further analysis only to manually examined examples. These limitations become more evident when trying to analyse the role of BGCs at an ecological scale. BiGSLICE could help overcome these problems by offering a new approach for a user-defined granularity classification of BGC, and thus facilitating function assignment.

One of the key points in the work is the approach to transform BGCs into vectors, a process that is detailed with precision and makes good sense. But the authors seem to overlook other (perhaps more straight forward) vectorisation methods such as BioVec (10.1371/journal.pone.0141287) or Pfam2vec (10.1093/nar/gkz654). The use of such approaches could reduce the dimensions of the vectors and potentially speed up the actual clustering step. Furthermore, Pfam2vec authors have shown that averaging the vectors of proteins in BGCs provides useful representations for clustering.

I recommend that this paper be accepted after minor revision.

Minor revisions about the software:

1) Input folder: Even though the wiki page about the input folder is very useful, a downloadable example of an input folder will make it very easy to test and users can familiarize with the output of the tool.

2) README file (Quick start / 3. Fetch the latest HMM models): The name of the script in the instruction

is "download_bigslice_hmmdb.py" while the installed script does not have the ".py" extension.
3) The minimum information that a cluster's genbank file must contain to be processed must be provided. Meaning, which features/annotations should the file have?. This will be helpful when an alternative tool to antiSMASH is used to detect BGCs, or in cases where the cluster borders are manually refined.

Level of Interest

Please indicate how interesting you found the manuscript: Choose an item.

Quality of Written English

Please indicate the quality of language in the manuscript: Choose an item.

Declaration of Competing Interests

Please complete a declaration of competing interests, considering the following questions:

- Have you in the past five years received reimbursements, fees, funding, or salary from an organisation that may in any way gain or lose financially from the publication of this manuscript, either now or in the future?
- Do you hold any stocks or shares in an organisation that may in any way gain or lose financially from the publication of this manuscript, either now or in the future?
- Do you hold or are you currently applying for any patents relating to the content of the manuscript?
- Have you received reimbursements, fees, funding, or salary from an organization that holds or has applied for patents relating to the content of the manuscript?
- Do you have any other financial competing interests?
- Do you have any non-financial competing interests in relation to this paper?

If you can answer no to all of the above, write 'I declare that I have no competing interests' below. If your reply is yes to any, please give details below.

I declare that I have no competing interests

I agree to the open peer review policy of the journal. I understand that my name will be included on my report to the authors and, if the manuscript is accepted for publication, my named report including any attachments I upload will be posted on the website along with the authors' responses. I agree for my report to be made available under an Open Access Creative Commons CC-BY license (http://creativecommons.org/licenses/by/4.0/). I understand that any comments which I do not wish to be included in my named report can be included as confidential comments to the editors, which will not be published.

Choose an item.

To further support our reviewers, we have joined with Publons, where you can gain additional credit to further highlight your hard work (see: https://publons.com/journal/530/gigascience). On publication of this paper, your review will be automatically added to Publons, you can then choose whether or not to claim your Publons credit. I understand this statement.

Yes Choose an item.
