# Supplementary material for: BiG-SLiCE: A highly scalable tool maps the diversity of 1.2 million biosynthetic gene clusters
Source: Gigascience. 2021 Jan 13;10(1):giaa154. doi: 10.1093/gigascience/giaa154 (PMC7804863; doi:10.1093/gigascience/giaa154)
Supplement: giaa154_Reviewer_2_Report_Original_Submission — Georg Zeller -- 10/20/2020 Reviewed [file giaa154_reviewer_2_report_original_submission.pdf]

Reviewer Report

Title: BiG-SLiCE: A Highly Scalable Tool Maps the Diversity of 1.2 Million Biosynthetic Gene Clusters

Version: Original Submission **Date:** 10/20/2020

Reviewer name: Georg Zeller

Reviewer Comments to Author:

Summary:
Kautsar et al. present BiG-SLICE, a software framework for large-scale analysis of biosynthetic gene clusters (BGCs) centered around a scalable clustering method (taken from scikit-learn) for the construction of gene cluster families (GCFs). Compared to a previous tool from the same group, called BiG-SCAPE, it comes with some trade-offs between scalability and clustering "accuracy", as is openly acknowledged by the authors.
An interesting part of their new method is the construction of feature sets for BGC clustering, i.e. domain subsets and increased-resolution sub-domains characteristic for BGCs and specific BGC classes. We're convinced that this will be useful beyond the proposed BiG-SLICE framework.
Finally, the authors present an application of their tool to create a global map of BGC diversity, which is unprecedented in scale (containing >1 mio BGCs), and perform further analyses to explore known and novel BGC diversity across prokaryotic and fungal genomes.
Overall the presented tool has the potential to help mine the deluge of microbial genomes (and MAGs) for fascinating and biomedically/-technologically relevant secondary metabolites as is nicely showcased by the accompanying analyses. The manuscript is logically structured, well written and contains effective display items. With some revisions, it absolutely deserves publication.
Our only major reservations concern the software, its design choices, documentation and the envisioned user group as detailed below.
Major concerns
1. When we tried to use the BiG-SLICE software, we encountered a few issues which raised some broader questions about usability and the target user group:
   a. BiG-SLICE is quite rigidly designed around input data coming (exclusively) from AntiSMASH (only seems to accept antismash-modified GenBank files); we could not get it to accept more standard GenBank files or ones generated by other tools such as DeepBGC.
   b. BiG-SLICE results are all stored in the SQL database and export is not straightforward. Programmatic access to the database is not (well) documented either.â€¨A more flexible design for BiG-SLICE, in particular export of results as flat-text files for easy import to enable quick follow-up analysis would be highly desirable. I feel that while GUI access may be most important for tools such as AntiSMASH, most users interested in really large-scale BGC analyses will (have to) possess more advanced computational skills and would therefore likely value programmatic access over graphical outputs. As a side note, I'd encourage the authors to consider more light-weight designs for future versions of BiG-SLICE as these may also scale better. Fig 5A to me suggests that further gains in efficiency (necessary for ever larger analyses of MAGs etc) cannot be expected from algorithmic

improvements (clustering currently only takes ~10% of the whole runtime), but from improved software engineering (e.g. for leaner IO).

2. We encourage the authors to extend documentation and fix issues with linked content on github:

a. https://github.com/medema-group/bigslice: hyperlink to pre-processed result on ~1.2M microbial BGCs from the NCBI database produces 404

b. https://github.com/medema-group/bigslice/wiki/Input-folder: GTDB-toolkit and NCBI taxonomy script hyperlinks produce 404

c. https://github.com/medema-group/bigslice/wiki/Program-parameters: program parameters are not documented

d. The `bigslice --version` command does not print the version.â€¨

Minor

Page 1. The sentence "Due to the sheer size of microbial and enzymological biodiversity, there exists a vast repertoire of potentially useful compounds remains to be unearthed" needs rewording.

Page 5. Fig 2C: Separation between distributions could be quantified by receiver-operating-characteristic (ROC) curves (also applies to Fig 3). Using a varying distance cutoff, pairs from the same class above the cutoff are counted as true-positives, below the cutoff as false negatives; pairs from different classes above the cutoff as false positives and as true negatives below the cutoff.

Fig 2D: consider changing scale to 0-100 for "percent" identity, as I am assuming this is referring to 0-100% and not 0-1.0%.

Page 13. Why is the T = 1100, established to be optimal in Fig 3A, not included among the thresholds tested here?

Page 14. Why was k = 500 chosen for k-means clustering?

Is the longish explanation for interpretation of non-deterministic k-means clustering needed? Why not stick to hierarchical clustering down to the 120K GCFs instead?

Page 15. Fig 6: Why aren't fungal clades clustered together (despite mentioned distinctions from prokaryotic clades)? Dendrogram in the middle is a bit too small to be used for interpretation.

Page 18. Fig 7: Consider labeling the x-axis (not just in the caption)

Level of Interest

Please indicate how interesting you found the manuscript: Choose an item.

Quality of Written English

Please indicate the quality of language in the manuscript: Choose an item.

Declaration of Competing Interests

Please complete a declaration of competing interests, considering the following questions:

- Have you in the past five years received reimbursements, fees, funding, or salary from an organisation that may in any way gain or lose financially from the publication of this manuscript, either now or in the future?
- Do you hold any stocks or shares in an organisation that may in any way gain or lose financially from the publication of this manuscript, either now or in the future?
- Do you hold or are you currently applying for any patents relating to the content of the manuscript?
- Have you received reimbursements, fees, funding, or salary from an organization that holds or has applied for patents relating to the content of the manuscript?
- Do you have any other financial competing interests?
- Do you have any non-financial competing interests in relation to this paper?

If you can answer no to all of the above, write 'I declare that I have no competing interests' below. If your reply is yes to any, please give details below.

I declare that I have no competing interests

I agree to the open peer review policy of the journal. I understand that my name will be included on my report to the authors and, if the manuscript is accepted for publication, my named report including any attachments I upload will be posted on the website along with the authors' responses. I agree for my report to be made available under an Open Access Creative Commons CC-BY license (http://creativecommons.org/licenses/by/4.0/). I understand that any comments which I do not wish to be included in my named report can be included as confidential comments to the editors, which will not be published.

Choose an item.

To further support our reviewers, we have joined with Publons, where you can gain additional credit to further highlight your hard work (see: https://publons.com/journal/530/gigascience). On publication of this paper, your review will be automatically added to Publons, you can then choose whether or not to claim your Publons credit. I understand this statement.

Yes Choose an item.
